# Healthy Patients With *AKR1D1* Mutation Not Requiring Primary Bile Acid Therapy: A Case Series

**DOI:** 10.1097/PG9.0000000000000372

**Published:** 2023-10-09

**Authors:** Akihiko Kimura, Jun Mori, Anh-Hoa Nguyen Pham, Kim-Oanh Bui Thi, Hajime Takei, Tsuyoshi Murai, Hisamitsu Hayashi, Hiroshi Nittono

**Affiliations:** From the * Department of Pediatrics, Kumamoto-Ashikita Medical Center for the Severely Disabled, Kumamoto, Japan; † Junshin Clinic Bile Acid Institute, Tokyo, Japan; ‡ Department of Pediatrics, Graduate School of Medical Sciences, Kyoto Prefectural University of Medicine, Kyoto, Japan; § Hepatology Department, National Children’s Hospital, Hanoi, Vietnam; ∥ Faculty of Pharmaceutical Science, Health Science University of Hokkaido, Hokkaido, Japan; ¶ Laboratory of Molecular Pharmacokinetics, Graduate School of Pharmaceutical Science, The University of Tokyo, Tokyo, Japan.

**Keywords:** primary bile acid therapy, *AKR1D1*, disorders of bile acid synthesis, Δ^4^-3-oxosteroid 5β-reductase deficiency

## Abstract

Δ^4^-3-Oxosteroid 5β-reductase (*AKR1D1*) deficiency typically causes severe cholestasis occurs in newborns, leading to death unless patients are treated with primary bile acids. However, we encountered an *AKR1D1* deficiency patient treated with only ursodeoxycholic acid who had cholestasis until about 1 year of age but then grew up healthy without further treatment. We also have been following other healthy patients with *AKR1D1* mutation who have never developed cholestasis and have not been treated. However, reports are few, involving 3 patients. To better understand and clinically manage a diverse group of patients with *AKR1D1* mutation who do not develop potentially fatal cholestasis in the neonatal period, ongoing accumulation and study of informative cases is needed.

**What Is Known**

- Infants with Δ^4^-3-oxosteroid 5β-reductase (*AKR1D1*) deficiency typically show rapid progression to cirrhosis without early diagnosis and initiation of primary bile acid treatment. Delay is likely to lead to cirrhosis, and liver failure requiring transplantation.
- Timely oral primary bile acid therapy for these infants has produced good clinical results.

**What Is New**

- Some children carry an *AKR1D1* mutation but do not have progression of cholestasis, remaining or becoming healthy without primary bile acid therapy.
- Variability of clinical course may reflect differences between patients involving the *AKR1D1* gene and/or other genes.

## INTRODUCTION

Δ^4^-3-oxosteroid 5β-reductase (*AKR1D1*) deficiency first was reported by Setchell et al. (1). In 2003, genetic analysis by Lemonde et al. (2) identified the responsible gene as *AKR1D1 (SRD5B1*), located on chromosome 7q32-33. Serum assays in *AKR1D1* deficiency show normal or slightly elevated concentrations of total bile acids (TBA) and γ-glutamyltransferase (GGT), but notable elevations of conjugated bilirubin and alanine aminotransferase (ALT). Stools are fatty, while pruritus is absent. During the biosynthesis of bile acids from cholesterol, decreased activity of *AKR1D1* enzymes interferes with the biosynthesis of primary bile acids while increasing the biosynthesis of unusual bile acids, specifically 3-oxo-Δ^4^ bile and allo-bile acids. Accordingly, this enzyme deficiency is diagnosed based on a combination of clinical symptoms, laboratory findings, gene analysis, and bile acid analysis. In most affected infants, the disease progresses to cirrhosis and liver failure if untreated, requiring liver transplantation. Patients found to have this disorder can be treated effectively with early initiation of primary bile acid therapy (3).

We report a 17-year-old Japanese girl with *AKR1D1* deficiency who had received not primary bile acid therapy but was treated in infancy with an earlier therapeutic agent, ursodeoxycholic acid (UDCA), until 12 months of age, with improvement of liver function test results. No ill effects occurred after UDCA was discontinued. How this patient with *AKR1D1* mutation avoided severe cholestasis during and after UDCA monotherapy is unclear. We offer a hypothesis that could explain how she regained and retained essentially normal liver function without primary bile acid therapy.

In addition, we studied 3 Vietnamese siblings who shared a *AKR1D1* mutation, including a 2-year-old boy with cholestasis since birth who is undergoing primary bile acid therapy with chenodeoxycholic acid (CDCA). In contrast, his 8-year-old sister and 10-year-old brother have shown no jaundice or liver dysfunction since birth. Neither of them ever received primary bile acid therapy. We carried out bile acid analysis in these 3 children as well as in our 17-year-old patient.

In this report, we consider how some patients with *AKR1D1* mutation might require primary bile acid therapy.

## METHODS

### Case Presentation

#### A 17-Year-Old Japanese Girl

This Japanese girl was born by spontaneous vaginal delivery without complications at 39 weeks of gestational age. Weight at birth was 2770*g*. From the age of 4 weeks, jaundice progressively worsened until 3 months of age, when she became deeply icteric and passed pale stools. Initial liver function test results were direct bilirubin (DBil), 8.3 mg/dL; GGT, 76 IU/L; ALT, 679 IU/L; and TBA, 2.7 μmol/L. UDCA therapy (5 mg/kg/day) was started immediately, and liver function test results gradually improved. However, excessive 3-oxo-Δ^4^ bile acids were detected in urine (Table 1), so we recommended primary bile acid therapy for the treatment of a suspected bile acid synthetic defect. When the parents declined that change in treatment, we increased the dose of UDCA to 10 mg/kg/day. A liver biopsy specimen obtained at 8 months of age showed giant cell transformation with bridging fibrosis and ductular proliferation, and we continued to detect excessive 3-oxo-Δ^4^ bile acids in urine during UDCA therapy.

**TABLE 1.** Bile acid analyses in an insufficiently treated Japanese girl with *AKR1D1* deficiency

	During UDCA treatment		Bile acid treatment stopped at 12 months of age	
	(8 months old)	(12 months old)	(16 years old)	(17 years old)
Serum (μmol/L)				
Cholic acid	Not detected	0.1	Trace	Not detected
Chenodeoxycholic acid	Not detected	0.7	1.3	0.7
Ursodeoxycholic acid	2.9	Not detected	Not detected	Not detected
Allo-bile acids	Not detected	4.4 (44.9%)	0.2 (3.8%)	Not detected
3-Oxo-Δ^4^ bile acids	5.6 (65.9%)	2.4 (24.4%)	3.7 (69.8%)	1.8 (56.3%)
Others	Trace	2.2	0.1	ND
Total bile acids	8.5	9.8	5.3	3.2
Urine (μmol/mmol creatinine)				
Cholic acid	0.7	2.4	Trace	Trace
Chenodeoxycholic acid	0.7	0.1	Not detected	0.1
Ursodeoxycholic acid	29.3	Not detected	Trace	Trace
Allo-bile acids	Not detected	0.3	0.1 (0.3%)	Not detected
3-Oxo-Δ^4^ bile acids	133.6 (79.0%)	107.4 (94.1%)	30.6 (97.1%)	18.6 (98.9%)
Others	4.9	3.9	0.8	0.1
Total bile acids	169.2	114.1	31.5	18.8

Allo-bile acids: allo-cholic acid and allo-chenodeoxycholic acid. 3-Oxo-Δ^4^ bile acids: 7α, 12α-dihydroxy-3-oxo-4-cholenoic acid and 7α-hydroxy-3-oxo-4-cholenoic acid. Bile acid analysis employed gas chromatography-mass spectrometry (GC-MS). ND = not detected; UDCA = ursodeoxycholic acid.

Despite these pathologic and biochemical findings, the patient has been in apparent good health without any treatment since 1 year of age. Liver function values also have been normal; current results are DBil, 0.2 mg/dL; GGT, 9 IU/L; ALT, 8 IU/L; and TBA, 1.6 μmol/L. Results of serum and urinary bile acid analyses are summarized in Table 1. Genetic testing by targeted next-generation sequencing detected compound heterozygosity in *AKR1D1* (c.378 + 2 T > C, c.668 G > A) (4). Initial testing had found only 1 heterozygous mutation in the *AKR1D1* gene (5). Despite the patient’s favorable present course, unusual bile acids such as 3-oxo-Δ^4^ bile acids remain detectable in serum and urine (Table 1). For reference, Supplemental Digital Content Table 1, http://links.lww.com/PG9/A138 shows normal values for bile acids.

#### Three Vietnamese Siblings

The 3 siblings referred to us from Vietnam were born to the same parents. Age and sex were 2 years (male; diagnosed at 6 months), 8 years (female), and 10 years (male). The parents had no significant medical history and were nonconsanguineous. We hospitalized the youngest sibling because of cholestatic jaundice and pale stool at 6 months of age. Liver function test results at the time of admission were DBil, 5.3 mg/dL; ALT 108 IU/L; and GGT 34 IU/L. The patient has mild coagulation disorder with a prothrombin time of 18.6s and an International Normalized Ratio of 1.52. Other laboratory abnormalities included tests hypoalbuminemia (2.6 g/dL). As urinary bile acid analysis for this infant detected large amounts of 3-oxo-Δ^4^ bile acids in the urine (Table 2), AKR*1D1* deficiency was suspected. Genetic analysis detected a mutation of *AKR1D1* (homozygous c.797G>A), so *AKR1D1* deficiency was diagnosed. CDCA replacement therapy (5 mg/kg/day) was begun immediately, and cholestasis gradually improved. All liver function results became normal a few months after initiation of CDCA therapy and have remained normal during 40 months of follow-up.

**TABLE 2.** Liver function tests and bile acid analyses in 3 Vietnamese siblings with *AKR1D1* deficiency

	1	2	3
	Affected 2-year-old boy	Healthy 8-year-old girl	Healthy 10-year-old boy
	During CDCA therapy	No liver dysfunction	No liver dysfunction
Total bilirubin (mg/dL)	0.2	0.5	0.5
Direct bilirubin (mg/dL)	Not done	0.1	0.1
AST (U/L)	36	24	26
ALT (U/L)	54	14	11
GGT (U/L)	16	13	12
Serum (μmol/L)			
Usual bile acids	Not done	7.0	25.8
3-Oxo Δ^4^ bile acids	Not done	1.0 (12.5%)	2.0 (7.3%)
Other bile acids	Not done	Not detected	Not detected
Total bile acids	Not done	8.0	27.8
Urine (μmol/mmol Cr)			
Usual bile acids	6.2	0.5	0.3
3-Oxo Δ^4^ bile acids	11.9 (64.1%)	19.2 (96.9%)	7.1 (94.6%)
Other bile acids	0.5	0.1	0.1
Total bile acids	18.6	19.8	7.5

Usual bile acids: cholic acid, chenodeoxycholic acid, ursodeoxycholic acid, deoxycholic acid, and lithocholic acid. 3-Oxo Δ^4^ bile acids: 7α,12α-dihydroxy-3-oxo-4-cholen-24-oic acid and 7α-hydroxy-3-oxo-4-cholen-24-oic acid. Dried filter paper urine was used for urinary bile acid analysis (6). AST = aspartate aminotransferase; ALT = alanine aminotransferase; GGT = γ-glutamyltransferase.

For the purposes of genetic counseling and diagnostic screening, we performed clinical examinations, liver function tests, and *AKR1D1* gene mutation analysis for other members of that patient’s family. Neither sibling of the 2-year-old showed any clinical manifestation of *AKR1D1* deficiency at any age, and all liver function test results were completely normal. Nonetheless, genetic analysis found both siblings to have the sane homozygous mutation that was present in the 2-year-old.

#### CDCA Therapy Suspension for 1 Month in a Japanese 2-Year-Old

We also cared for a 2-year-old Japanese boy with *AKR1D1* deficiency (compound heterozygous, c.797 G > A, c.580-14 A > G) whose CDCA therapy we suspended for 1 month; based on our experience with Japanese patients, we reasoned that after CDCA therapy (5 mg/kg/day) had successfully averted cholestasis and its complications during neonatal and infantile periods, further CDCA might not be required (7). His results are shown below together with those for the other children.

## RESULTS

In our patient whose parents permitted only UDCA therapy, this treatment appeared sufficient for the prevention of progressive liver damage, and no ill effects were evident even after UDCA was stopped at the end of infancy. This patient is currently healthy. In *AKR1D1* deficiency, 3-oxo-Δ^4^ bile acids typically accumulate in hepatocytes during the neonatal period and early infancy, leading to chronic cholestasis and consequent cirrhosis. Primary bile acid therapy exerts negative feedback upon *CYP7A1*, suppressing biosynthesis of potentially toxic 3-oxo-Δ^4^ bile acids. UDCA therapy does not offer this benefit (8). Why UDCA therapy proved sufficient in this patient is not clear. Liver damage appears to have resolved spontaneously despite absence of CDCA treatment.

In the Vietnamese family with 3 siblings showing an *AKR1D1* mutation, 2 remained healthy without primary bile acid therapy. According to their parents, neither untreated child has had any episodes of jaundice or liver dysfunction since birth—even though we found percentages of 3-oxo-Δ^4^ bile acids in urine to be elevated (Table 2).

In our Japanese patient with a 1-month suspension of CDCA therapy at 2 years of age, neither DBil nor ALT showed elevations (Supplemental Digital Content Table 1, http://links.lww.com/PG9/A138). The most abundant usual bile acid in serum during CDCA therapy had been CDCA. During CDCA therapy suspension for 1 month, small amounts of 3-oxo-Δ^4^ bile and allo-bile acids were detected in serum, as they were at the time CDCA therapy was initiated. During the month of suspension, percentages of 3-oxo-Δ^4^ bile and allo-bile acids among TBA gradually increased, from 4.2% and 5.2% to 29.9% and 34.3%, respectively (Supplemental Digital Content Table 2, http://links.lww.com/PG9/A138). This increased percentage of unusual bile acids mostly reflected a decrease in concentration of usual bile acids (mostly CDCA) during that month, although amounts of 3-oxo-Δ^4^ bile and allo-bile acids in urine showed some increases beyond those present during CDCA therapy. During the month without treatment, no deterioration in liver function or increase in serum TBA occurred. Although the serum concentration of CDCA decreased as expected, serum concentration of 3-oxo-Δ^4^ bile acids did not increase (7).

## DISCUSSION

Untreated infants with *AKR1D1* deficiency usually show rapid progression to cirrhosis unless in the absence of prompt clinical intervention. Diagnostic delay is likely to lead to liver failure requiring transplantation.

Cholestasis in this disease typically develops in neonates and infants, requiring primary bile acid therapy. Nonetheless some patients, like our 17-year-old Japanese patient, remain healthy since infancy without CDCA treatment. Palermo et al. (9) have reported a similar case (Table 3). In addition, cholestasis that continues from birth might not be seen, such as in the 2 untreated siblings from Vietnam. Variable courses are known to occur in other bile acid biosynthetic disorders, such as sterol 27-hydroxylase (*CYP27A1*) and oxysterol 7α-hydroxylase (*CYP7B1*) deficiency. In those conditions, severe cholestasis may occur in newborns or progressive neurologic disorders may develop in adolescents and adults (13–16).

**TABLE 3.** Patients with *AKR1D1* deficiency showing an atypical course

	Molecular defect	Clinical findings
Single child		
Present patient, a 17-year-old girl	c.378 + 2T > C and c.668 G > A	See the case presentation in this paper
Reported Zhang et al. (10)	c.797 G > A Heterozygous	Patient 5 in the referenced report. Cholestasis at 10 days of age, started UDCA therapy at 10 months and liver dysfunction completely resolved 5 months later. He remained healthy without further treatment
Reported Clayton et al. (2,9,11)	c.662 C > T Homozygous	Developed cholestasis and disturbed consciousness at age 3 weeks
		Received UDCA therapy without improvement. At 8 months, started on CA plus CDCA therapy. Over 3 months, liver function improved; not been treated since. Now 13 years old and in good health
Family cases		
3 Vietnamese siblings in our case	c.797 G > A Homozygous	See the case presentation in this paper
Reported Gonzales et al. (3,12)	c.467 C > G and c.668 G > A	Twin sisters with onset at 1 month. One with severe cholestasis hepatosplenomegaly ascites; refractory to CA therapy. The other’s liver dysfunction improved fairly early with CA therapy, including a liver function test. Both now doing well with CA therapy.

CA = cholic acid; CDCA = chenodeoxycholic acid; UDCA = ursodeoxycholic acid.

We suspect that 3-oxo-Δ^4^ bile acids, which are highly toxic to hepatocytes and cause liver damage, do not always accumulate in the hepatocytes. Excretion of 3-oxo-Δ^4^ bile acids into bile canaliculi, a vital physiologic function, is considered to be defective in these conditions (1). However, the ability of multidrug resistance-associated protein 3 (MRP3) to excrete 3-oxo-Δ^4^ bile acids from hepatocytes into sinusoidal blood may vary with age (17). Severity of liver damage in *AKR1D1* deficiency may reflect age dependence of MRP3 activity (Supplemental Digital Content Figure 1, http://links.lww.com/PG9/A137). However, this is only our hypothesis.

In a genetic analysis of *AKR1D1* deficiency in 5 patients, Drury et al. (18) raised an important point: while mutations in *AKR1D1* can cause bile acid deficiency and liver damage by compromising physiologic *AKR1D1* activity unless primary bile acid therapy is initiated early in life, adaptive response later in life may then reduce the need for treatment. We further believe that symptoms of this bile acid biosynthetic disorder might not wholly depend on mutations of *AKR1D1*, but also might be influenced by other genetic factors. Additionally, phenotypic expression may depend on the permeability of the gene in a given individual. All these factors can lead to variable timing and severity of symptoms among patients with the same mutation.

Severity of symptoms has been reported to vary for the same mutation (3). According to Clayton, the same mutation in *AKR1D1* in the same family can result in phenotypes ranging from death in infancy from liver failure to the absence of clinical liver disease in the fourth decade. Major questions persist about what determinants and mechanisms result in liver disease or its absence in the presence of *AKR1D1* mutations (19). In short, genetic diagnosis alone may be insufficient for predicting the likelihood and severity of liver disease or establishing the need for or the preferred mode of treatment.

Clinical variability among the Vietnamese siblings whom we confirmed to have an identical *AKR1D1* mutation also suggested that some patients may have high MRP3 activity even in the neonatal period. Thus, *AKR1D1* deficiency may have 2 different manifestations, such as fatal infantile progressive cholestasis or an adult-onset neurologic disorder (20). Such phenotypic variability also characterizes other abnormalities of genes that encode enzymes involved in oxysterol and bile acid biosynthesis, such as *CYP7B1* or *CYP27A1*. These genetic defects cause liver disease in some infants, but also may result in progressive central nervous system disease, polyneuropathy, and tendon xanthomas in order individuals (cerebrotendianous xanthomatosis) (20). The clinical course of the first patient described here showed yet another pattern: cholestasis during the neonatal period and subsequent good health. For the last patient in our series, a 2-year-old Japanese boy with *AKR1D1* deficiency, CDCA therapy (5 mg/kg/day) was suspended for 1 month considering his age. During this suspension, no deterioration in liver function or increase in serum TBA occurred. Although the serum concentration of CDCA decreased as we expected, concentrations of 3-oxo-Δ^4^ bile acids did not increase (Supplemental Digital Content Table 2, http://links.lww.com/PG9/A138) (7). The results of our trial of CDCA discontinuation appear to support our belief that growth and maturation may decrease the need for this treatment, at least in some individuals. This patient currently is doing well on CDCA therapy.

Unfortunately, these cases have a short follow-up period, and the patients will need to be monitored closely in the future. In addition, since liver function alone cannot fully evaluate clinical stability or therapeutic effect, liver pathologic findings after treatment would have much to contribute. These cases were evaluated histopathologically at diagnosis, but no follow-up liver biopsy was performed because the patient now is healthy with no liver dysfunction, and the family has not consented to a biopsy.

In conclusion, *AKR1D1* gene mutation is recognized as a major cause of defective bile acid biosynthesis. However, 3 of the 5 patients with an *AKR1D1* gene mutation in this case series had no liver dysfunction. Further investigation concerning details of the pathogenicity of this mutation, including additional long-term follow-up of healthy children and adults who are homozygous (or compound heterozygous) for the mutation, will be needed.

## ACKNOWLEDGMENTS

The authors would like to thank Dr Takao Togawa (Department of Pediatrics and Neonatology, Nagoya City University Graduate School of Medicine Sciences, Nagoya, Japan) and Dr Kazuo Imagawa (Department of Pediatrics, University of Tsukuba Hospital, Ibaraki, Japan) for genetic analysis. Informed consent for observations and analysis, such as bile acid analysis in serum and urine and gene analysis of *AKR1D1*, in this study, was obtained from parents of patients.

## Supplementary Material


