# Supplementary material for: Healthy Patients With AKR1D1 Mutation Not Requiring Primary Bile Acid Therapy: A Case Series
Source: JPGN Rep. 2023 Oct 9;4(4):e372. doi: 10.1097/PG9.0000000000000372 (PMC10684241; doi:10.1097/PG9.0000000000000372)
Supplement: Supplementary file 1 [file pg9-4-e372-s001.pdf]

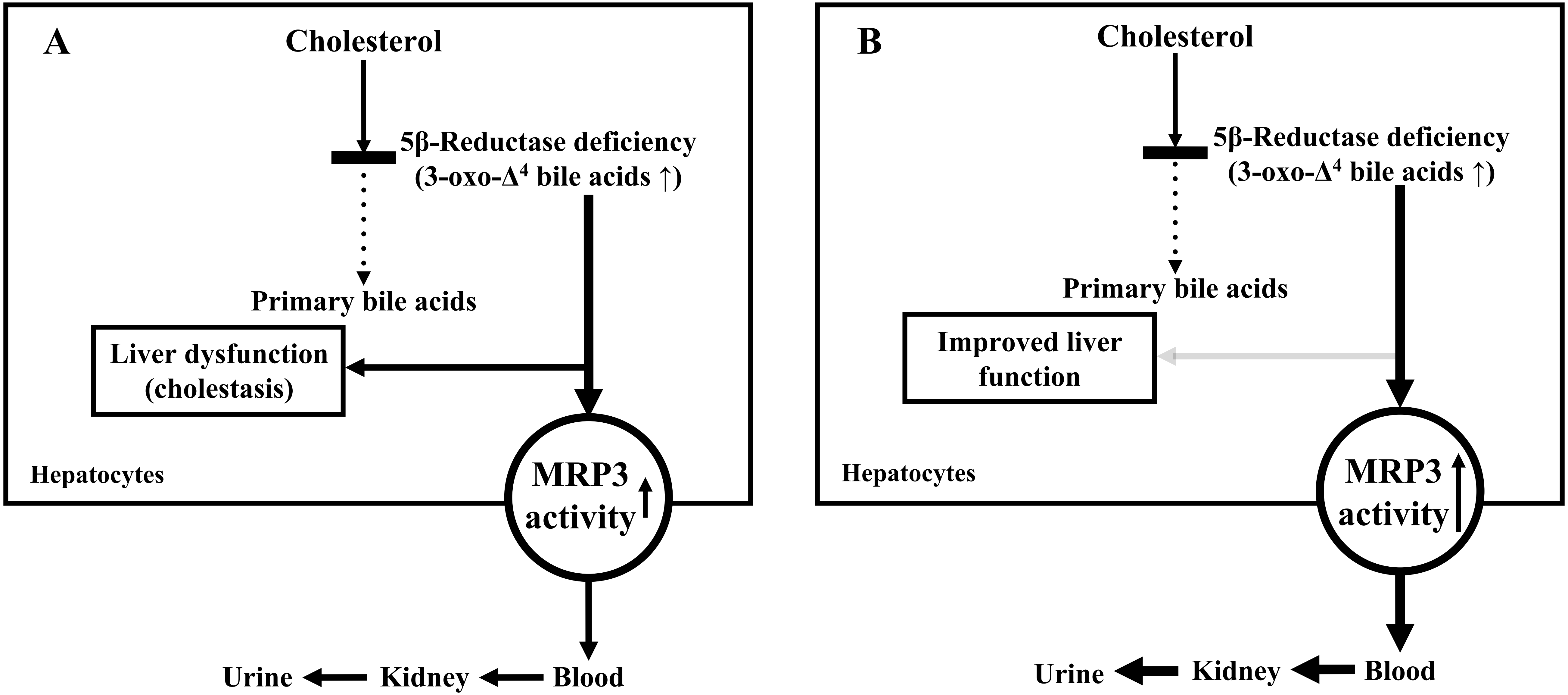
A
Cholesterol
5β-Reductase deficiency
(3-oxo-Δ4 bile acids ↑)
Primary bile acids
Liver dysfunction
(cholestasis)
MRP3
activity ↑
Hepatocytes
Urine ← Kidney ← Blood
B
Cholesterol
5β-Reductase deficiency
(3-oxo-Δ4 bile acids ↑)
Primary bile acids
Improved liver
function
MRP3
activity ↑
Hepatocytes
Urine ← Kidney ← Blood

Supplemental Figure 1. We hypothesized that age-dependent severity of liver damage in *AKR1D1* deficiency depends on variable multidrug resistance-associated protein 3 (MRP3) activity.

Panel A: In neonates and young infants with *AKR1D1* deficiency, MRP3 activity is low, impeding excretion of 3-oxo-Δ^4 bile acids from hepatocytes into sinusoidal blood. As a consequence, highly hepatotoxic 3-oxo-Δ^4 bile acids accumulate in hepatocytes, damaging these cells.

Panel B: Late in infancy, activity of MRP3 has increased. As a result, 3-oxo-Δ^4 bile acids are more readily excreted from hepatocytes into sinusoidal blood. 3-Oxo-Δ^4 bile acids within hepatocytes are greatly decreased, as are manifestations of liver damage such as jaundice, compromised hepatic function, and cholestasis. Unusual bile acids excreted from hepatocytes into the blood rapidly reach the kidneys and are excreted in urine, decreasing their serum concentrations.
