# Supplementary material for: Healthy Patients With AKR1D1 Mutation Not Requiring Primary Bile Acid Therapy: A Case Series
Source: JPGN Rep. 2023 Oct 9;4(4):e372. doi: 10.1097/PG9.0000000000000372 (PMC10684241; doi:10.1097/PG9.0000000000000372)
Supplement: Supplementary file 2 [file pg9-4-e372-s002.pdf]

Supplemental Table 1. Normal values for bile acids.

Serum (μ mol/L)		1y (N=5)	2y (N=4)	10-16y (N=7)	
CA		0.98±0.84	0.98±1.28	0.32±0.16	
CDCA		3.69±3.70	3.09±1.80	1.24±0.61	
UDCA		0.46±0.35	1.32±0.79	0.41±0.45	
Allo-bile acids		0.06±0.03	0.06±0.03	0.02±0.01	
3-Oxo-Δ4 bile acids		0.01±0.01	0.02±0.01	0.01±0.01	
Others		0.68±0.45	0.59±0.24	0.80±0.34	
TBA		5.90±5.18	6.05±3.78	2.96±1.56	
Urine (μmol/mmol Cr)	8m (N=6)	1y (N=20)	2y (N=26)	9-14y (N=5)	
CA	0.26±0.20	0.32±0.29	0.19±0.15	0.26±0.47	
CDCA	0.69±0.42	0.24±0.21	0.36±0.23	0.10±0.08	
UDCA	0.03±0.04	0.42±0.87	0.35±0.31	0.18±0.17	
Allo-bile acids	0.01±0.01	0.02±0.04	0.02±0.03	0.00±0.00	
3-Oxo-Δ4 bile acids	0.02±0.03	0.05±0.07	0.04±0.04	0.01±0.01	
Others	0.73±0.82	0.34±0.36	0.45±0.37	0.13±0.09	
TBA	1.71±1.32	1.39±1.26	1.41±0.	0.65±0.40	

CA, cholic acid; CDCA, chenodeoxycholic acid; UDCA, ursodeoxycholic acid; TBA, total bile acids; m, age in months; y, age in years.

Supplemental Table 2. Liver function tests and bile acid analyses in a patient with *AKR1D1* deficiency before and during CDCA treatment suspension at 2 years of age

	Before CDCA treatment suspension	During CDCA treatment suspension	
		After 2 weeks	After 4 weeks
Total bilirubin (mg/dL)	0.2	0.2	0.3
Direct bilirubin (mg/dL)	0.1	0.1	0.1
AST (U/L)	29	29	28
ALT (U/L)	13	12	12
Serum (μmol/L)			
Usual bile acids	23.8	5.2	1.5
Allo-bile acids	1.1 (4.2 %)	1.6 (18.9%)	1.4 (29.9 %)
3-Oxo Δ^4 bile acids	1.4 (5.2 %)	1.7 (19.7 %)	1.6 (34.3 %)
Other bile acids	0.1	0.1	0.1
Total bile acids	26.4	8.6	4.7
Urine (μmol/mmol Cr)			
Usual bile acids	1.7	0.5	0.2
Allo-bile acids	0.3	0.2	0.2
3-Oxo Δ^4 bile acids	37.1 (93.6 %)	30.6 (96.4)	33.7 (97.4%)
Other bile acids	0.5	0.5	0.4
Total bile acids	39.6	31.7	34.6

AST, aspartate aminotransferase; ALT, alanine aminotransferase. Usual bile acids: cholic acid, chenodeoxycholic acid, ursodeoxycholic acid, deoxycholic acid, and lithocholic acid. Allo-bile acids: allo-cholic acid and allo-chenodeoxycholic acid. 3-Oxo Δ^4 bile acids: 7α,12α-dihydroxy-3-oxo-4-cholen-24-oic acid and 7α-hydroxy-3-oxo-4-cholen-24-oic acid.
